# Optimizing football analytics: Dimensionality reduction meets machine learning for offensive and defensive player profiling

**DOI:** 10.1371/journal.pone.0353239

**Published:** 2026-09-09

**Authors:** Rodrigo Yáñez-Sepúlveda, Aldo Vásquez-Bonilla, Rodrigo Olivares, Pablo Olivares, Guillermo Cortés-Roco, Eduardo Guzmán-Muñoz, Marcelo Tuesta, Jorge Olivares-Arancibia, Boryi A. Becerra-Patiño, Juan David Paucar-Uribe, Diego Andrés Rada-Perdigón, Exal Garcia-Carrillo, Tomás Rivera-Kofler, Vicente Javier Clemente-Suárez, José Francisco López-Gil

**Affiliations:** 1 Faculty of Education and Humanities, Universidad Andres Bello, Viña del Mar, Chile; 2 Department of Sport Sciences, Faculty of Sport and Health Sciences, Fit Generation Research Institute, Andorra la Vella, Andorra; 3 Universidad de Extremadura, Facultad de Ciencias del Deporte, Caceres, España; 4 Escuela de Ingeniería Informática, Universidad de Valparaíso, Valparaíso, Chile; 5 Facultad de Ciencias de la Vida, Universidad Viña del Mar, Viña del Mar, Chile; 6 Escuela de Kinesiología, Facultad de Salud, Universidad Santo Tomás, Talca, Chile; 7 Escuela de Kinesiología, Facultad de Ciencias de la Salud, Universidad Autónoma de Chile, Talca, Chile; 8 Exercise and Rehabilitation Sciences Laboratory, School of Physical Therapy, Faculty of Rehabilitation Sciences, Universidad Andres Bello, Santiago, Chile; 9 Grupo AFySE, Investigación en Actividad Física y Salud Escolar, Escuela de Pedagogía en Educación Física, Facultad de Educación, Universidad de Las Américas, Santiago, Chile; 10 Faculty of Physical Education, National Pedagogical University, Bogotá, Colombia; 11 Department of Physical Activity Sciences, Faculty of Education Sciences, Universidad Católica del Maule, Talca, Chile; 12 School of Education, Faculty of Human Sciences, Universidad Bernardo O'Higgins, Santiago, Chile; 13 Faculty of Medicine, Health and Sports, Universidad Europea de Madrid, Madrid, Spain; 14 Grupo de Investigación en Cultura, Educación y Sociedad, Universidad de la Costa, Barranquilla, Colombia; 15 School of Medicine, Universidad Espíritu Santo, Samborondón, Ecuador; 16 Vicerrectoría de Investigación y Postgrado, Universidad de Los Lagos, Osorno, Chile; University of Illinois Urbana-Champaign, UNITED STATES OF AMERICA

## Abstract

**Background:**

This study focuses on the quantitative analysis of football players’ performance based on a set of performance data.

**Objective:**

The objective of this study was to develop a multivariate analytical model to identify, quantify, and predict the most relevant factors that determine the offensive and defensive profiles of professional football players, thereby optimizing functional segmentation, performance analysis, and decision-making processes in the contexts of scouting and data-based tactical planning.

**Materials and methods:**

A dataset was compiled through automated extraction (web scraping) via the Python programming language and its specialized libraries. The full dataset comprised 2,689 player–league records corresponding to 2,530 unique players from Europe’s five major UEFA-affiliated national leagues during the 2022–2023 season (159 players who transferred between two leagues mid-season contributed one record per league, 2,530 + 159 = 2,689). After applying a minimum-eligibility filter of >450 minutes played to ensure stable per-90 metrics, 1,624 player–league records (1,620 unique players) were retained for the modeling workflow. Dimensionality reduction techniques (PCA) and explainable machine learning (Shapley Additive exPlanations [SHAP]) with predictive modeling were applied. Eight algorithms were trained and compared to predict PC1 Offensive and PC1 Defensive.

**Results:**

The First Offensive Principal Component (PC1 Offensive) synthesizes actions that generate scoring opportunities, such as actions prior to shots (SCA_p90, 13.3%), progressive receptions (RecProg_p90, 13.0%) and total shots (Shots_p90, 12.7%). The first-defensive principal component (PC1 Defensive) highlights tackles (Tkl_p90, 19.1%), blocks (15.6%) and interceptions (15.6%).

**Conclusions:**

Finally, the inclusion of SHAP allows for an in-depth interpretation of the models, identifying the key statistical factors that influence players’ offensive and defensive contributions, which is valuable for scouting, tactical analysis and player development.

## Introduction

Football is considered the most popular sport in the world, with approximately 270 million active players and an even larger fan base [1,2]. This sport is characterized by a dynamic combination of aerobic endurance, anaerobic bursts of intensity, and the development of character strength [3–5]. The complexity of the game stems from its multiple demands: technical skills (ball control, passing accuracy), tactical intelligence (positional awareness, decision-making) and physical skills (accelerations, decelerations and high-intensity sprints) [6–8].

Football has been described as a complex, dynamic and nonlinear system in which the confrontation between two teams depends on constant adaptation to technical-tactical, situational and contextual factors that are constantly changing [9]. These elements interact in a highly unpredictable environment, where quick decisions under fatigue determine competitive outcomes [10,11]. Modern football has also evolved into a highly competitive environment in which elite clubs seek marginal gains in nutrition, psychology and biomechanics to optimize performance [12–14]. This unpredictability of the game demands an effective response to the cooperation–opposition environment [15].

In recent years, the integration of artificial intelligence (AI) techniques has revolutionized the analysis of tactical behavior in football, offering new opportunities in multiple facets of the game. The creation of science and data analysis departments in clubs reflects this trend, applying methodologies such as artificial neural networks, deep learning and machine learning (ML) [16,17]. ML can process large volumes of information in real time, extracting patterns imperceptible to manual analysis, and providing advantages in decision-making on strategies, line-ups and training, as well as in injury prediction and performance optimization [18,19].

The availability of football big data and its integration have generated a more comprehensive view of the factors that influence performance: physical, physiological [20] and technical-tactical [21]. This integrated perspective provides a better understanding of the interdependence between individual and collective performance, intra- and intercoordination training, playing style and tactical-strategic nuances [20,22]. AI-based tactical behavior mapping is particularly promising for improving player development, refining training strategies and increasing the overall level of match analysis [23].

In this context, studies such as those by Taylor et al. [24] reported that the tactical component is composed of interdependent situational and notational variables, reporting multiple ball-related metrics in elite teams. In addition, numerous analyses have addressed aspects such as match states, ball possession, opponent analysis [25], game conditions (winning, losing or drawing) and match locations [26]. These types of variables directly affect offensive and defensive conditions [27], whose influence varies according to the tactical role assumed by the player [28]. In fact, several authors argue that scientific research should delve deeper into the role of play to generate more robust conclusions via advanced methodologies [29].

Thus, the interaction between technical-tactical and statistical variables favors the development of predictive models of the game [30], making it necessary to move toward more sophisticated approaches to strategic profiling in professional footballers. The use of expectation maximization algorithms to automate formations [31], spatiotemporal kernels to identify scoring opportunities [32,33], or multiscale techniques to predict recurring offensive sequences [34] shows that statistical and computational methods can automate and even predict real tactical situations in football [16].

Consequently, the growing technical demands of football require more advanced analytical methods to extract meaningful information from large volumes of data [35–37]. Recent research has emphasized the need to integrate tactical metrics with wellness indicators (sleep, nutrition, biomarkers), creating holistic player profiles [38,39]. This framework has been enhanced by AI and ML, which allow for the discovery of imperceptible patterns through dimensionality reduction and explainable techniques [18,19].

Therefore, this study aimed to develop a multivariate analytical model that integrated dimensionality reduction (PCA) and explainable ML (Shapley Additive exPlanations [SHAP]) to identify, quantify and predict the most relevant factors that determine the offensive and defensive profiles of professional footballers, optimizing functional segmentation, performance analysis and decision-making processes in the contexts of scouting and data-driven tactical planning.

## Methods

### Design and sample

The dataset was compiled through automated web extraction via Python and specialized libraries [40]. After data cleaning and preprocessing, the compiled dataset comprised 2,689 player–league records and 124 variables, corresponding to 2,530 unique professional players competing in the five major UEFA-affiliated European leagues during the 2022–2023 season. The 159 records that exceed the number of unique players correspond to players who transferred between two of the five leagues during the season and therefore contribute one record per league (2,530 + 159 = 2,689). The distribution of records across leagues was: Ligue 1, 565; LaLiga, 550; Serie A, 544; Premier League, 540; and Bundesliga, 490 (total = 2,689). The modeling unit is the player–league record, and the player-grouped train/test split assigns all records of a given player to the same partition. To ensure stable per-90 metrics, players who did not play more than 450 minutes were excluded; after this minimum-eligibility filter (>450 minutes played), 1,624 player–league records (1,620 unique players) entered the modeling workflow. The four records exceeding the number of unique players in this analytic subset correspond to transferred players who surpassed the 450-minute threshold in both of their leagues (1,620 + 4 = 1,624).

### Ethical considerations

Although the dataset comprises exclusively publicly available information and contains no personally identifiable or sensitive data beyond the publicly known identities of the players as professional athletes, all procedures strictly adhered to the ethical principles outlined in the Declaration of Helsinki [41]. The study design ensured full respect for the dignity, rights, and autonomy of all individuals indirectly involved. The research was conducted in accordance with international ethical standards governing the responsible use of observational data in sports science and human performance research.

### Data analysis

#### Data acquisition and preprocessing.

1. **Data Loading:** We begin by loading a dataset (presumably a CSV file) into a Pandas DataFrame. The initial dimensions of the DataFrame are (2689 rows, 124 columns).
2. **Initial inspection:** A basic inspection is performed to understand the data structure, including the data types (.info()), descriptive statistics (.describe()), and visualization of the first rows (.head()). Numeric (float64, int64) and categorical (object) variables are identified.
3. **Coding of Categorical Variables:** Categorical variables (e.g., Nation, Pos, Squad, Comp) were transformed into numerical representations using one-hot encoding.
4. **Feature Scaling:** Numeric features are scaled to ensure that they all have a comparable range (crucial for PCA and some regression models) via StandardScaler, which applies standardization.

$$\begin{array}{c} z=\frac{x-\mu }{\sigma } \end{array}$$

5 Missing data were handled through a structured preprocessing workflow. Rows with missing values for the target variables were excluded from the corresponding supervised task. For model predictors, numeric variables were imputed using the median, whereas categorical variables were imputed using an explicit “Unknown” category. After imputation, the data were checked again to ensure that no missing predictor values remained before model fitting.

#### Non-circularity and leakage control.

To prevent circularity between the constructed indices and the supervised models, the variables used to build each principal component were excluded from the predictor set of the corresponding model. The offensive PCA was built from Goals_p90, Shots_p90, SoT_p90, G/Sh, G/SoT, Assists_p90, PasAss_p90, PPA_p90, CrsPA_p90, PasProg_p90, SCA_p90, GCA_p90, CarProg_p90 and RecProg_p90; the defensive PCA from Tkl_p90, Int_p90, Blocks_p90, Clr_p90, Tkl + Int_p90, TklW_p90, TklDef3rd, TklMid3rd and TklAtt3rd. All remaining numeric and contextual variables (position, league, age, minutes, passing, carrying, aerial, touch-location and discipline metrics) served as predictors. The train/test split was performed before any data-dependent step; median imputation, standardization (StandardScaler) and PCA were fitted on the training partition only and applied unchanged to the test partition. An automated audit confirmed zero overlap between PCA-construction variables and predictors; this audit operated at the level of the exact index-defining variables. Some base counterparts of these indices and subcomponents of composite metrics nonetheless remained among the predictors — notably tackles won (TklWon), the base equivalent of TklW_p90; blocked passes and blocked shots, which compose Blocks_p90; and ScaPassLive, ScaSh, ScaDrib and GcaPassLive, which compose the SCA/GCA families. Together with the strong mutual correlation among elite-player metrics, this partial structural overlap helps explain the high R² values and the prominence of TklWon in the defensive SHAP ranking, and reinforces our interpretation of the supervised stage as a consistency and interpretability check rather than as evidence of independent predictive validity. Overall predictor missingness in the processed dataset was 0.0%, as count statistics with structural zeros were coded as zero at source.

#### Exploratory Data Analysis (EDA).

Univariate analysis: Histograms and box plots were used to visualize the distribution of each numerical variable.

- **Bivariate and Multivariate Analysis:**
  - Scatter plots to explore relationships between pairs of variables.
  - Correlation heatmaps were used to visualize the Pearson correlation matrix, which helps identify multicollinearity and variables strongly related to the possible target variables.

$$\begin{array}{c} =\frac{\sum_{i=\text{1}}^{n}\left(x_{i}-\overset{―}{x}\right)\left(y_{i}-\overset{―}{y}\right)}{\sqrt{\sum_{i=\text{1}}^{n}{\left(x_{i}-\overset{―}{x}\right)}^{\text{2}}}\sqrt{\sum_{i=\text{1}}^{n}{\left(y_{i}-\overset{―}{y}\right)}^{\text{2}}}} \end{array}$$

### Dimension reduction: Principal component analysis (PCA)

Owing to the high dimensionality (124 columns), PCA is applied to reduce the number of variables while preserving as much variance as possible.

a **Calculation of the covariance matrix on the standardized data.**
b **Resolution of eigenvalues and eigenvectors:**

$$\begin{array}{c} \Sigma w_{j}=\lambda_{j}w_{j} \end{array}$$

**Formation of main components:** ${\text{PC}}_{k}=w_{k\text{1}}Z_{\text{1}}+w_{k\text{2}}Z_{\text{2}}+\ldots +w_{kp}Z_{p}$

Component Selection: The first k components that explain, for example, ≥ 95% of the cumulative variance are retained.

Practical application: Two aggregate components, PC1 Offensive and PC1 Defensive, were derived from the principal component analysis and subsequently used as target variables for the predictive models.

### Predictive modeling

Eight regression algorithms were trained and compared: random forest, support vector regression (SVR), ridge regression, linear regression, decision tree, k-nearest neighbors, ElasticNet, and LASSO regression. Model evaluation was based on a player-grouped 80/20 train/test split, in which all observations of a given player were assigned to a single partition to avoid information leakage from repeated players (1,624 player–league records for 1,620 unique players after the > 450-minute eligibility filter); a random 80/20 split was additionally computed as a sensitivity analysis. Because a single hold-out split can yield optimistic estimates, R² values are reported to two decimal places; generalizability was additionally assessed with repeated player-grouped k-fold cross-validation (5 folds × 2 repeats), across which random forest reached R² = 0.91 ± 0.01 (offensive) and 0.92 ± 0.01 (defensive), confirming the hold-out results. Hyperparameters were fixed and pre-specified (tree models: max_depth = 10, min_samples_split = 10; random forest: n_estimators = 200; random seed = 42; linear models used default regularization), with no automated hyperparameter search. Performance was assessed via the coefficient of determination (R²), mean absolute error (MAE), mean squared error (MSE), and root mean square error (RMSE). The random forest model was selected for SHAP-based interpretation because, among the models capable of capturing non-linear interactions, it attained a high coefficient of determination on a single player-grouped hold-out split (offensive R² ≈ 0.91; defensive R² ≈ 0.93), comparable to the regularized linear models (Ridge ≈ 0.93 and ≈ 0.94). Because elite-player metrics are mutually correlated and playing-time covariates contribute strongly, these values are interpreted as a consistency and interpretability check on the PCA-derived indices rather than as evidence of independent predictive validity; a tree-based model was chosen because SHAP attribution is most informative for non-linear ensembles. Once random forest was identified as the reference model, the SHAP method was applied.

**Data** division: 80% training/20% test.

- Model training: Each algorithm is adjusted with numerical (scaled) and categorical (coded) characteristics.
- Evaluation:

**Table:** 

Metrics	Score	Interpretation
MAE	$\text{MAE}=\frac{\text{1}}{n}\sum_{i=\text{1}}^{n}\left|y_{i}-{\hat{y}}_{i}\right|$	Average absolute error
MSE	$\text{MSE}=\frac{\text{1}}{n}\sum_{i=\text{1}}^{n}{\left(y_{i}-{\hat{y}}_{i}\right)}^{\text{2}}$	Penalize major errors more severely
RMSE	$\text{RMSE}=\sqrt{\text{MSE}}$	Square root of the MSE
R²	$R^{\text{2}}=\text{1}-\frac{{\text{SS}}_{\text{res}}}{{\text{SS}}_{\text{tot}}}$	Proportion of variance explained

To avoid conceptual overlap, the manuscript now explicitly distinguishes the variables used to construct the PCA-derived indices from the predictors used in the supervised modeling stage.

### Model interpretation with SHAP

To understand the influence of each feature on the predictions, SHAP is used, which assigns each variable a value of importance on the basis of game theory: $\varphi_{j}=\sum_{S\subseteq F\backslash \{j\}}\frac{|S|!\left(|F|-|S|-\text{1}\right)!}{|F|!}\left(\text{val}\left(S\cup \{j\}\right)-\text{val}(S)\right)$

- **SHAP summary graphs:** overall importance.
- **Dependency graphs:** reveal how the value of a feature affects the prediction.
- **Force plots:** explain individual predictions, highlighting which features push the prediction up or down.

## Results

**Fig 1:**
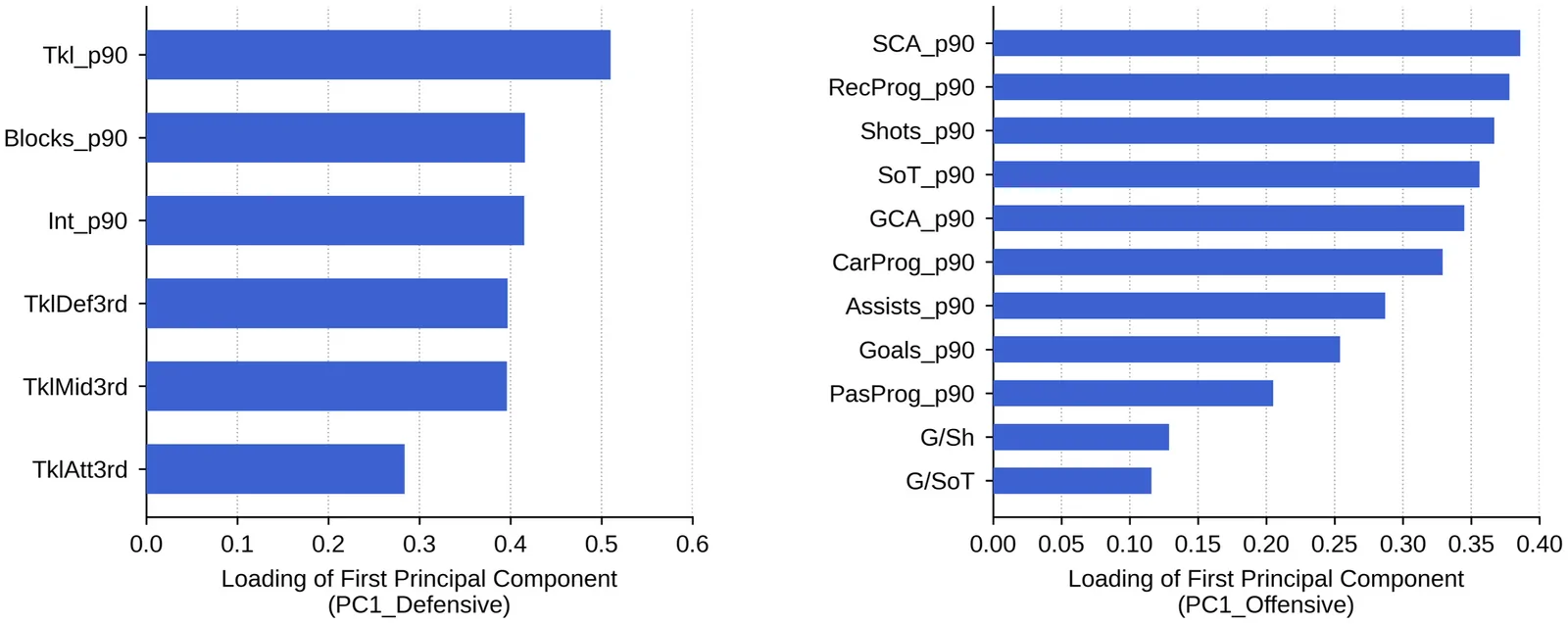
Main offensive and defensive components.

Principal component analysis reveals that the first principal component-offensive (PC1 Offensive) synthesizes a robust construct of offensive volume and implication, where the variables with the greatest weight correspond to actions that generate goal opportunities, such as actions prior to shots (SCA_p90, 13.3%), progressive receptions (RecProg_p90, 13.0%) and total shots (Shots_p90, 12.7%). These, together with shots on goal, actions prior to goals and progressive dribbles, make up a profile of a player highly involved in actively generating play, collectively explaining approximately 75% of the component's variance. In contrast, efficiency metrics such as goals per shot (G/Sh) have reduced loads, indicating that the component does not reflect effectiveness but rather offensive prominence. The first principal component (PC1 Defensive) integrates a synthetic index of defensive commitment, where tackles (Tkl_p90, 19.1%), blocks (15.6%) and interceptions (15.6%) stand out, together with their spatial distributions in the different thirds of the field. This configuration allows us to capture both the intensity and location of defensive actions, providing a valuable analytical tool for multidimensional profiling of players according to their style, tactical role and contributions in both phases of the game (Fig 1).

The bivariate representation of the offensive (PC1 Offensive) and defensive (PC1 Defensive) principal components enables a visual and integrated classification of players based on their functional profile, utilizing individual performance metrics. Each point represents a player, coded by position and minutes played, located in a space where the X-axis reflects their involvement in offensive actions (shots, assists, progressions), and the Y-axis reflects their involvement in defensive tasks (interceptions, tackles, blocks).

**Fig 2:**
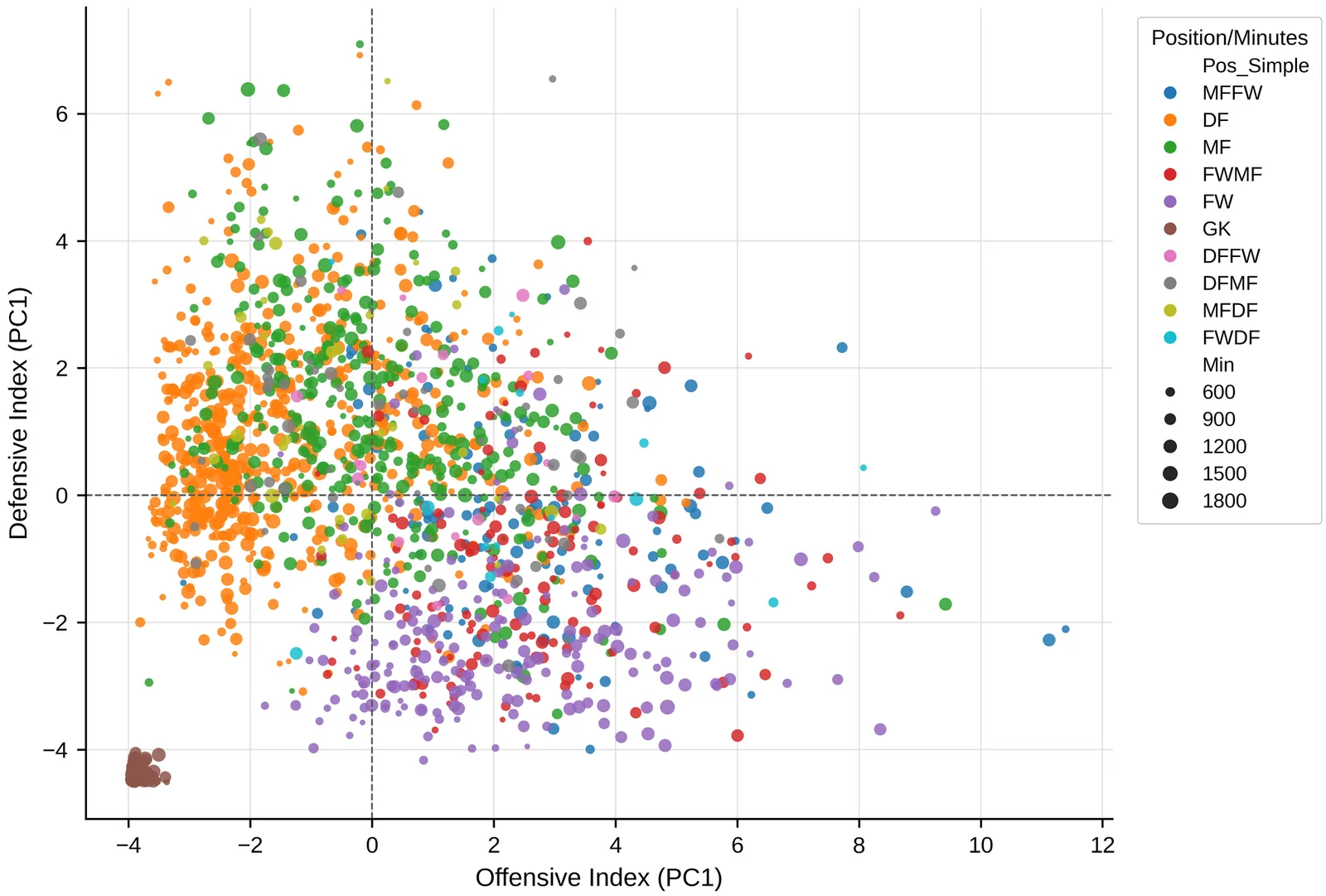
Offensive and defensive indices.

The distribution reveals a concentration around the central offensive axis, indicating balanced profiles, and allows specific areas to be identified: the upper right quadrant groups together complete players with high activity in both phases, the lower right quadrant groups together attackers with low defensive commitment, the upper left quadrant groups together defenders with little offensive impact, and the lower left quadrant groups together players with low overall participation. This approach facilitates tactical analysis, role segmentation, and decision-making in scouting and strategic planning processes from a multivariate perspective (Fig 2).

Table 1 presents a comparative evaluation of eight regression algorithms used to predict players’ defensive and offensive contributions in professional football based on principal component scores derived from technical and tactical performance metrics. For each domain (Defensive and Offensive), three performance metrics are reported: the coefficient of determination (R²), mean absolute error (MAE), and root mean square error (RMSE). The random forest model reproduced the PCA-derived offensive and defensive indices with a high coefficient of determination on the single player-grouped hold-out split (offensive R² ≈ 0.91, MAE = 0.53, RMSE = 0.78; defensive R² ≈ 0.93, MAE = 0.42, RMSE = 0.64), comparable to the regularized linear models (Ridge ≈ 0.93 and ≈ 0.94). Because the metrics that define the indices are mutually correlated and playing-time covariates (minutes, 90s) contribute strongly, these values should be read as a consistency and interpretability check rather than as substantive predictive validity. Random Forest was retained as the reference model for SHAP interpretation given its ability to capture non-linear interactions. It performed competitively against all the other models in both the defensive (R² ≈ 0.93) and offensive (R² ≈ 0.91) domains, with low overall error values, comparably to the regularized linear models. The regularized linear models attained the highest scores (ridge and linear regression, R² ≈ 0.93 offensive and ≈ 0.94 defensive), marginally above random forest. Support vector regression was intermediate (R² ≈ 0.84 in both domains), followed by the decision tree (R² ≈ 0.83 offensive and ≈ 0.85 defensive). ElasticNet and k-nearest neighbors showed lower explanatory power (R² ≈ 0.72–0.75 and ≈ 0.66–0.72, respectively), whereas Lasso performed worst (R² ≈ 0.53 offensive and ≈ 0.57 defensive), with correspondingly higher error metrics. These findings support the use of interpretable ensemble models, such as random forest, for the SHAP-based analysis of football performance: random forest matched the predictive accuracy of the regularized linear models while additionally enabling the analysis of non-linear feature contributions. Its combination of competitive accuracy and interpretability makes it well suited to modeling the complex, nonlinear patterns associated with functional contribution in elite football.

**Table 1:** Comparative performance of machine learning models for predicting defensive and offensive indices.

Model	Defensive			Offensive		
	R²	MAE	RMSE	R²	MAE	RMSE
**Ridge**	0.94	0.43	0.57	0.93	0.51	0.69
**Linear Regression**	0.94	0.43	0.58	0.93	0.52	0.69
**RandomForest**	0.93	0.42	0.64	0.91	0.53	0.78
**SVR**	0.85	0.55	0.93	0.84	0.58	1.06
**Decision Tree**	0.85	0.62	0.92	0.83	0.71	1.11
**ElasticNet**	0.72	0.92	1.26	0.75	0.94	1.31
**K Neighbors**	0.68	0.89	1.35	0.72	0.85	1.41
**Lasso**	0.57	1.21	1.57	0.53	1.36	1.82

Table note: the Offensive and Defensive columns report performance for the PC1_Offensive and PC1_Defensive prediction tasks, respectively. The previously included “Total” column was undefined and merely duplicated the Offensive values; it has been removed and no aggregate score is defined. All reported values were recomputed from the leakage-controlled, player-grouped pipeline and are given to two decimal places. Generalizability was confirmed by repeated player-grouped k-fold cross-validation (5 folds × 2 repeats): Random Forest R² = 0.91 ± 0.01 (offensive) and 0.92 ± 0.01 (defensive), with ridge and linear regression at R² ≈ 0.93 in both domains. Note: SVR: support vector regression; K Neighbors: K-nearest neighbors (KNN); R2: R-square; MAE: mean absolute error; RMSE: root mean square error.

**Fig 3:**
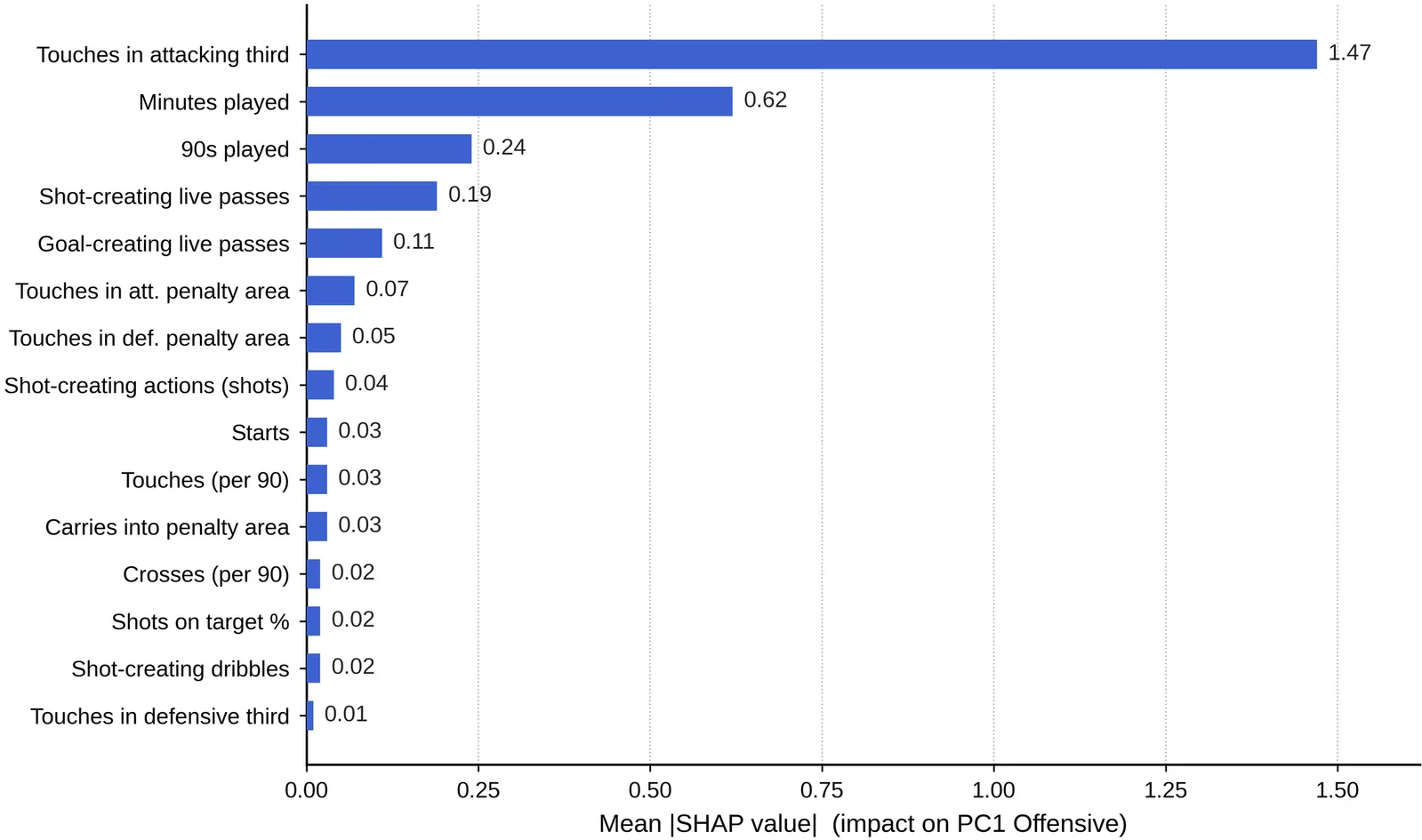
Contribution of offensive metrics to the machine learning model via SHAP.

Fig 3 shows the relative importance of the offensive predictors for reproducing the main offensive component (PC1 Offensive), estimated as the mean absolute SHAP (SHapley Additive exPlanations) value over the player-grouped test set. Because the index-defining variables (goals, shots, shots on target and the remaining PCA inputs) were excluded from the predictor set, this ranking reflects only non-circular contributors and therefore differs from raw goal-scoring output. The dominant feature by a wide margin is touches in the attacking third (num__TouAtt3rd, mean |SHAP| ≈ 1.47), indicating that sustained presence in the final third is the strongest proxy for offensive involvement. Playing-time volume follows (minutes played ≈ 0.62; 90s played ≈ 0.24), consistent with the interpretation that the supervised stage largely captures involvement and exposure rather than independent determinants. The next contributors are chance-creation actions—shot-creating live-ball passes (num__ScaPassLive ≈ 0.19) and goal-creating live-ball passes (num__GcaPassLive ≈ 0.11)—together with touches in the attacking penalty area (num__TouAttPen ≈ 0.07) and, to a lesser degree, shot-creating actions from shots (≈ 0.04), starts, touches per 90 and carries into the penalty area (each ≈ 0.03). The remaining variables (crosses per 90, shots-on-target percentage, shot-creating dribbles and touches in the defensive third) contribute marginally (≤ 0.02). Once finishing volume is removed, the offensive index is thus attributed primarily to attacking-third occupation and chance creation.

**Fig 4:**
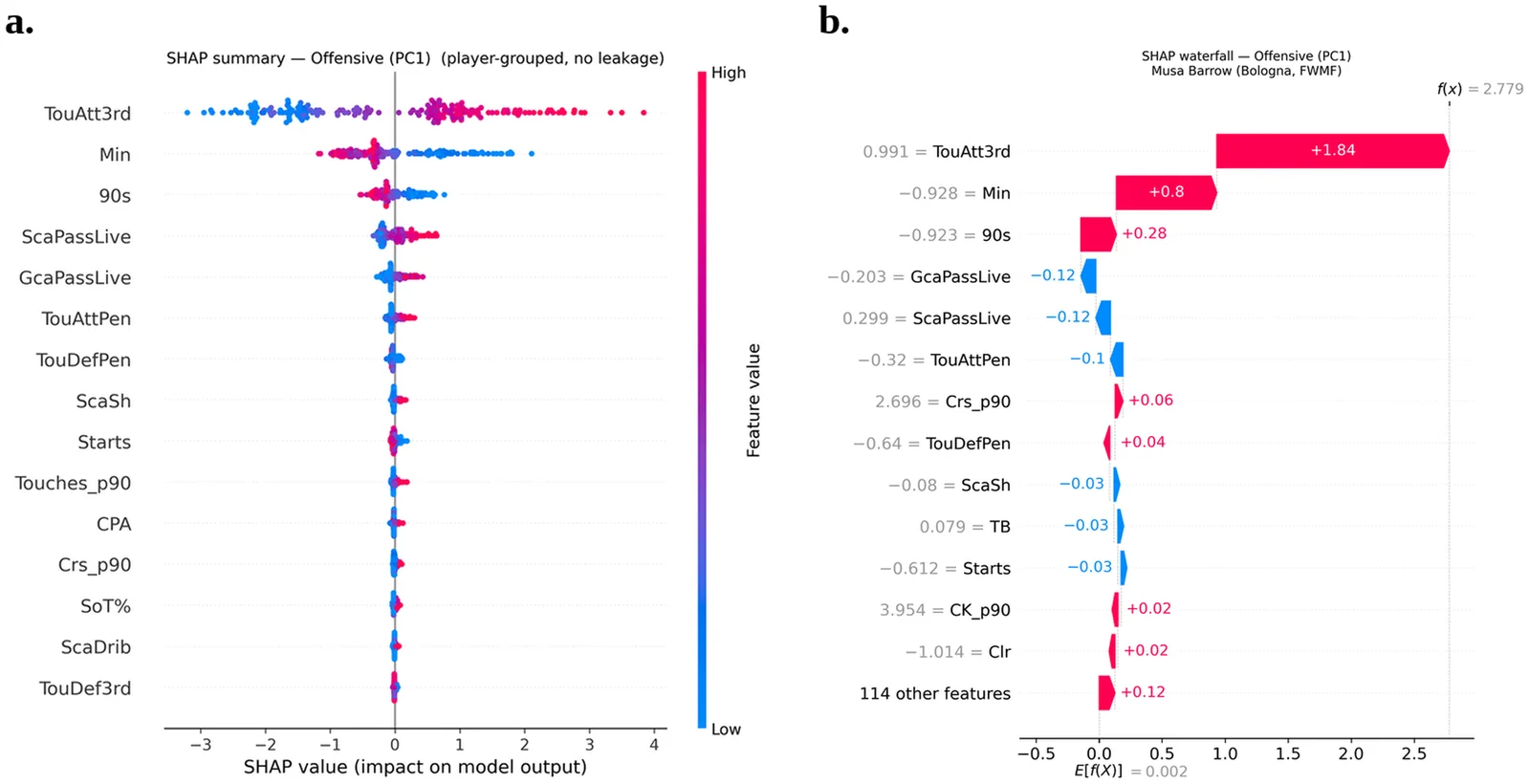
SHAP model prediction at the offensive level.

The interpretability of the model is enhanced by complementary SHAP visualizations that allow predictions to be broken down and contextualized. In Fig 4a, the beeswarm summary shows, for each player match, how each feature influences the model's prediction: points to the right represent a positive impact, whereas those to the left reflect a decrease in the prediction. The color encodes the magnitude of the original value of the variable (blue = low, fuchsia = high), and the horizontal dispersion highlights the variability of the impact according to the individual context of each observation, revealing the nonlinear interaction between variables. Fig 4b presents a waterfall diagram for a specific prediction, breaking down how the base value of the model is adjusted through blocks representing the contributions of each characteristic. The blue blocks indicate a reduction in the prediction, and the fuchsia blocks indicate an increase, allowing us to explicitly visualize how non-circular factors such as num__TouAtt3rd, num__Min, or num__GcaPassLive modify the result. This granular representation is crucial for comprehending the model's logic at the individual level and validating its consistency in practical applications.

**Fig 5:**
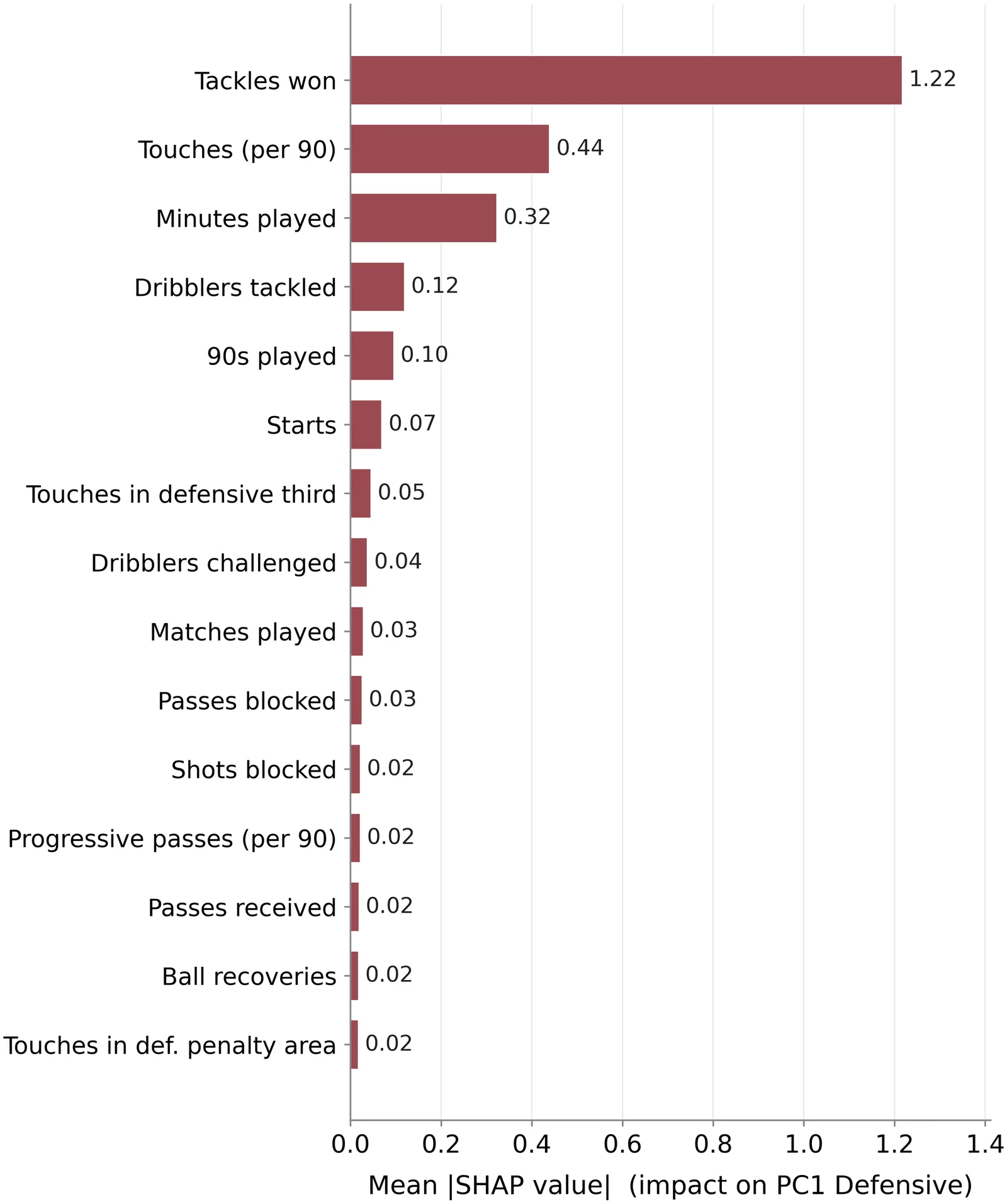
Contribution of defensive metrics to the machine learning model via SHAP.

Fig 5 shows the SHAP-based importance of the defensive predictors for reproducing PC1 Defensive, again computed as the mean absolute SHAP value over the player-grouped test set after excluding the index-defining defensive variables. Tackles won dominate the ranking (num__TklWon, mean |SHAP| ≈ 1.22), followed by overall ball involvement (touches per 90 ≈ 0.44) and playing-time volume (minutes played ≈ 0.32; 90s played ≈ 0.10). Direct defensive duels contribute next—dribblers tackled (num__TklDri ≈ 0.12) and dribblers challenged (num__TklDriAtt ≈ 0.04)—together with starts (≈ 0.07) and touches in the defensive third (≈ 0.05). Blocking actions (passes blocked ≈ 0.03; shots blocked ≈ 0.02), progressive passing volume, passes received and ball recoveries (each ≈ 0.02) complete the leading contributors. As with the offensive model, this profile indicates that the defensive index is driven chiefly by tackling success and defensive ball involvement, with playing-time covariates contributing substantially, once the index-defining tackle, interception and block counts are removed; the supervised stage is therefore best read as a consistency and interpretability check rather than as evidence of independent determinants (Fig 5).

**Fig 6:**
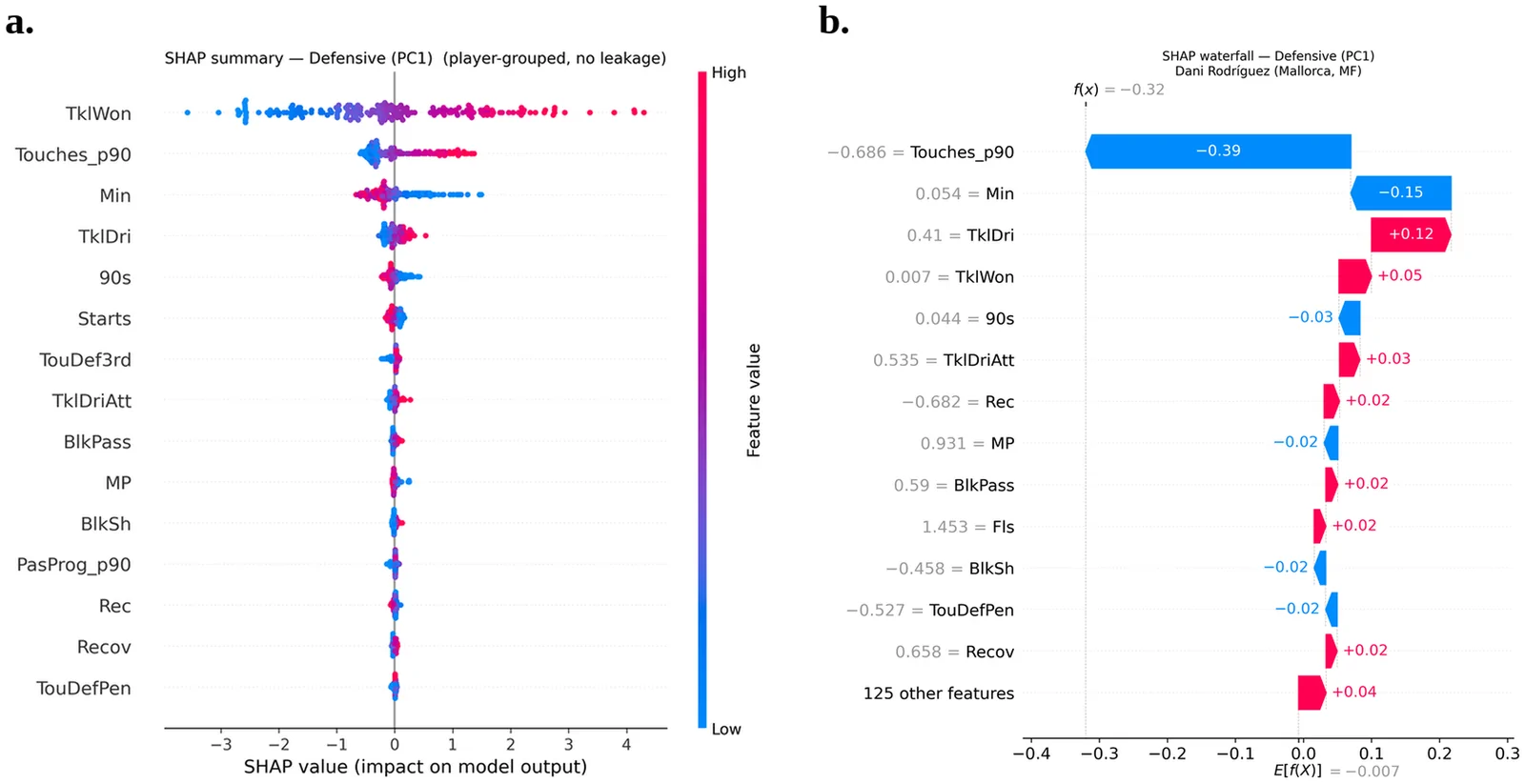
SHAP model prediction at the defensive level.

SHAP's ‘beeswarm’ diagram for defensive assessment. Each point represents a player match; the X-axis reflects the influence (positive or negative) on the prediction. The color codes the value of the characteristic (blue = low, fuchsia = high). For example, high values of tackles won and overall ball involvement (fuchsia) shift the prediction toward higher defensive scores (right side), whereas low values (blue) pull it toward lower scores. The width of each cloud shows how much variability each metric produces among different players (Fig 6).

Waterfall for a specific observation. It starts at the base value of the model. E[f(X)]and adds subtractions from each variable until the final prediction is reached.*f*(*x*)=0.32. The blue blocks lower (improve) the rating, and the fuchsia blocks raise it (worsen it). In this case, high overall touch involvement (num__Touches_p90) and minutes played lower this midfielder’s predicted defensive index (blue), whereas tackles of dribblers (num__TklDri) and tackles won (num__TklWon) raise it (fuchsia).

## Discussion

The PCA allowed us to condense the offensive contribution into an axis that reflects volume and prominence rather than efficiency: higher loads in SCA_p90 (13.3%), RecProg_p90 (13.0%) and Shots_p90 (12.7%), whereas conversion indicators had less relative weight, suggesting that the component captures involvement in play generation and not necessarily the quality of that production.

This pattern is in line with recent developments that recommend evaluating actions in context (e.g., distance, pressure, previous sequence), given that metrics based solely on volume (shots, touches) can overestimate contributions without translating into actual goal expectations or tactical advantages. Along these lines, frameworks such as Valuing Actions by Estimating Probabilities (VAEP) propose assigning value to any action according to its probabilistic impact on the outcome, overcoming the limitations of traditional metrics focused on goals/shots. Integrating this logic of contextual value could complement the present offensive approach based on protagonism and enrich the practical reading of profiles [42].

The defensive axis emerged as a more important engagement index for tackles (Tkl_p90, 19.1%), blocks (15.6%) and interceptions (15.6%), highlighting the location of the actions (thirds of the field), with particular relevance of the middle third in the model explanations (e.g., TklMid3rd in SHAP). This is consistent with studies that, using tracking data and explainable ML, successfully predict ball recoveries based on pressure, interline distances and numerical superiority, highlighting that transition windows in mid-zones account for much of the tactical defensive effectiveness [43].

In the comparison of algorithms, the random forest algorithm demonstrated, in the leakage-controlled player-grouped analysis, R² ≈ 0.91 (offensive) and R² ≈ 0.93 (defensive), performing comparably to the regularized linear models across the offensive and defensive domains; it was retained for SHAP-based interpretation because it captures the non-linear interactions inherent in the game rather than because it outperformed the linear and SVR approaches. This result is consistent with recent reviews of the field, where tree-based ensembles tend to excel in tabular event data because of their ability to model nonlinear relationships and interaction effects inherent in the game, especially when combined with tactical feature engineering. Furthermore, the use of SHAP provides transparency by breaking down the marginal contributions of variables—a growing requirement for the practical adoption of ML in technical bodies—and aligning inferences with interpretable tactical criteria [18,19,44].

The bivariate embedding of PC1 Offensive × PC1 Defensive allowed us to segment functional roles (e.g., ‘complete’ vs. ‘offensive/defensive specialists’) and, with SHAP, explain the levers that drive each profile. This logic fits well with role-conscious and multidimensional frameworks such as PlayeRank, which have proven useful for ranking and comparing athletes based on thousands of positionally aware events. The practical translation involves (i) setting thresholds by position/role and (ii) monitoring longitudinal drifts of the player in the PCA plane along with SHAP alerts (e.g., a decrease in RecProg or Tkl + Int) [45].

This approach reinforces the trend toward data-based models for evaluating tactical behavior while highlighting the limitations of relying solely on events when team structure (spaces, distances, densities) modulates offensive/defensive success. The literature review indicates that the greatest potential emerges when integrating domains (sports science + computer science) and tracking data with events, suggesting the incorporation of spatial metrics (areas, compactness, superiority) in the no possession and transition phases as a natural extension [46].

Finally, the findings support the use of ensemble-based models such as random forest for multifactorial predictive tasks in football performance analysis. These models combine competitive accuracy with interpretability when modeling complex, nonlinear patterns associated with functional contribution in elite football. However, limitations remain, such as the exclusive reliance on event data (without tracking), the absence of situational variables (home advantage, opponent quality, match status) and the focus on a single season, which may restrict temporal generalization. These limitations do not invalidate the findings, but they do point to future lines of research that incorporate multi-source data and adjustment for tactical and situational contexts.

### Practical applications and limitations

The characteristics of sport make football dynamic and constantly changing, which means that the metrics used, although abundant and detailed, may not fully capture specific and contextual elements of players and teams. Factors such as the game strategy, the coach's tactical style, or the specific characteristics of each competition can influence the interpretation of the data, limiting the possibility of generalizing the findings.

Furthermore, it is worth noting that the application of machine learning techniques, such as random forest, in the analysis of football performance is still relatively rare in scientific literature. This represents a limitation in terms of comparative references and external validation, as few studies have compared the results obtained or enriched the methodological discussion relative to previous approaches in the same field. Although a single train–test split can overestimate model performance, we mitigated this concern by performing repeated player-grouped k-fold cross-validation (5 folds × 2 repeats); random forest R² remained high and stable (offensive 0.91 ± 0.01; defensive 0.92 ± 0.01), indicating that the reported performance is not an artifact of a single partition.

We further note that, because elite-player performance metrics are mutually correlated, the supervised models retained high R² even after excluding the index-defining variables; playing-time covariates (minutes, 90s) also contributed to the offensive prediction. The supervised stage should therefore be interpreted as a consistency and interpretability analysis of the PCA-derived indices rather than as discovery of independent determinants, and the SHAP rankings are reported for the player-grouped, non-circular model.

The findings generated from the application of machine learning techniques can be extremely useful for football coaches and clubs in optimizing individual and collective performance and predicting matches, player line-ups and strategies against opposing teams. Indeed, future prospects may lie in this area, in the application of the elements provided by the model and their implementation with the teams analyzed.

The analytical workflow documented in this study represents a comprehensive analysis of football players’ performance. A combination of preprocessing, dimensionality reduction to create aggregate performance metrics (offensive and defensive PC1), and predictive modeling is used.

## Conclusions

This study successfully developed a multivariate analytical model integrating dimensionality reduction and explainable machine learning to identify, quantify, and predict the key factors determining offensive and defensive profiles in professional football players. The PC1 Offensive synthesized actions that generate scoring opportunities, whereas PC1 Defensive emphasized defensive engagement metrics. Among eight compared algorithms, random forest reproduced the PCA-derived offensive and defensive indices with a high coefficient of determination on a player-grouped hold-out split, with closely matching repeated k-fold cross-validation estimates, and served as the basis for SHAP interpretation, with regularized linear models performing comparably; because this performance largely reflects involvement and volume covariates, it should be interpreted as a consistency and interpretability analysis rather than as evidence of independent predictive determinants.

From a practical perspective, the bivariate embedding of offensive and defensive components enables evidence-based functional segmentation of players, distinguishing complete contributors from specialized roles. Scouting departments and coaching staffs can leverage SHAP-derived explanations to understand the marginal contribution of specific metrics (e.g., progressive receptions, tackles in the middle third) to each player's profile, facilitating targeted recruitment and tactical planning.

Future research should integrate positional tracking data to capture off-ball movements, defensive positioning, and spatial-temporal dynamics. Longitudinal multi-season designs could assess profile stability and predict performance trajectories. Additionally, incorporating match contextual variables and cross-league comparisons would enhance external validity and practical applicability.
